# TP53 mutation status: emerging biomarker for precision radiation medicine?

**DOI:** 10.18632/oncoscience.468

**Published:** 2018-08-28

**Authors:** Henning Willers, Meng Wang, Cyril H. Benes

**Affiliations:** Department of Radiation Oncology, Massachusetts General Hospital, Harvard Medical School, Boston, Massachusetts USA

**Keywords:** TP53, PARP inhibitor, biomarker, reactive oxygen species, radiosensitization

Combinations of radiation therapy with molecular targeted agents and immune checkpoint inhibitors offer enormous potential for personalizing anti-cancer treatments and improving cure rates [1]. Predictive tumor biomarkers will be needed to optimally stratify patients for such combination treatment approaches. For combining radiation with targeted drugs, preclinical studies are, therefore, increasingly utilizing annotated tumor cell lines to model the inter-tumoral heterogeneity that is thought to impact treatment outcomes in the clinic [2, 3].

In a recent report, Liu et al. [4] sought to characterize the radiosensitizing properties of the PARP-1/2 inhibitor olaparib in a panel of 9 bladder cancer cell lines. Olaparib radiosensitized all of these cell lines but to varying extent, with dose enhancement factors ranging from 1.22 to 2.27. Interestingly, radiosensitization was correlated with the induction of potentially lethal DNA double-strand breaks (DSB) but not with RAD51 foci formation, suggesting that the sensitivity of cells to olaparib was not driven by a homologous recombination repair (HRR) defect. Increased levels of reactive oxygen species (ROS) were observed in olaparib-treated tumor cells as well as in tissue explants from bladder cancer patients. Unexpectedly, olaparib-induced DNA damage was found in cells residing in the G1 phase of the cell cycle, which was inconsistent with the classical paradigm of PARP inhibitor-mediated synthetic lethality due to a HRR defect in S-phase. Consistent with this observation, a ROS scavenger protected cells against olaparib. Additional experiments indicated that olaparib-induced ROS were required for the radiosensitizing effects of olaparib and originated from mitochondria. Because ROS and DSB were produced within only a few hours of olaparib treatment, a transcriptional mechanism of ROS regulation by PARP-1 seemed unlikely. The data are compatible with a direct function of PARP-1 affecting mitochondria of bladder cancer cells though this was not studied in greater detail.

**Figure 1:**
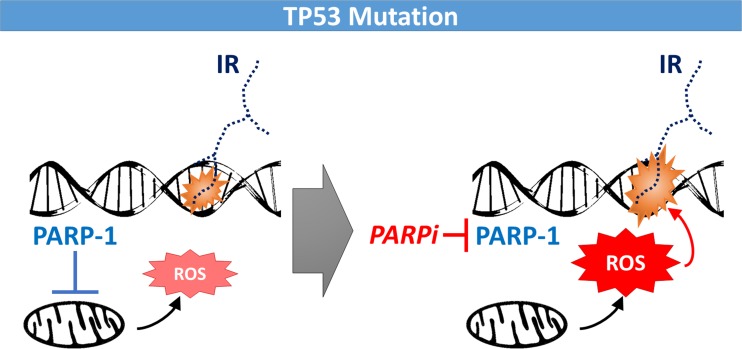
Model of PARP-1 function in TP53 mutated cancer cells In contrast to wild-type cells, cells with mutated TP53 require PARP-1 for suppressing reactive oxygen species (ROS) originating from mitochondria. Catalytic inhibition of PARP-1 by a PARP inhibitor (PARPi) leads to higher levels of ROS which interact with ionizing radiation (IR) to produce greater levels of lethal DNA damage.

With regard to predictive biomarkers of the radiosensitizing effect mediated by olaparib, mutation of the TP53 tumor suppressor was correlated with enhanced radiosensitization in the bladder cancer cell line panel [4]. This observation was confirmed in additional non-isogenic and isogenic cell line models, including lung and breast cancer. Loss of p53 function associated with increased PARP-1 expression in bladder cancer cell lines and tumors, supporting a model in which perturbed ROS regulation in cells with mutated TP53 require PARP-1 for survival in response to cytotoxic insults (Figure 1). This model is consistent with the emerging view of PARP-1 as a pleiotropic protein with multiple cellular functions including ROS control and p53's role in redox regulation [5]. It was suggested that an increase in ROS levels due to PARP-1 catalytic inhibition enhances the amount of DNA damage caused by a given dose of ionizing radiation, thereby increasing the likelihood of cell kill. However, additional mechanisms of ROS-mediated cell death may be in play as well. Also, which other PARP-1 functions contributed to the radiosensitizing effects of olaparib in this study remains to be fully elucidated. Notably, olaparib radiosensitized HRR-proficient cell lines as long as TP53 was mutated. In contrast, HRR-deficient cells with wild-type TP53 were poorly radiosensitized.

The role of TP53 in the radiation response has been studied by numerous investigators and cannot be reviewed here due to space constraints. However, what seems clear is that TP53 alone does not impact the cellular ability to repair lethal DSB caused by ionizing radiation [6]. Accordingly, TP53 status alone may have little clinical value as a predictive biomarker for radiation therapy. However, TP53 mutation may be a useful marker for stratifying patients who are treated with radiation plus a targeted radiosensitizer in certain settings [3, 4]. Furthermore, TP53 may have predictive (as well as prognostic) value when combined with other genomic factors – one example being KRAS mutation. Recent clinical and pre-clinical data indicate that KRAS/ TP53 double-mutant (KP) cancers are more resistant to radiation therapy than KRAS mutant but TP53 wild-type tumors [7, 8]. At the same time, the KP genotype exhibits specific molecular characteristics targeting of which may overcome the radiation resistance associated with it [7].

Lastly, pre-clinical discovery of genomic biomarkers for precision radiation medicine is limited by the number of tumor models used. Not surprisingly, the study by Liu et al. [4], which was based on 9 bladder cancer cell lines, was only able to identify genomic biomarkers with sufficiently high incidence (and magnitude of effect), i.e., TP53 mutation, which is a common alteration in many cancers. Linking less frequent genomic alterations in tumors to radiation resistance/sensitivity or radiosensitizing drug effects will require much larger numbers of annotated tumor cell lines and medium- to high-throughput screening approaches [2].
